# Giant Prolactinoma Causing Hydrocephalus and Intracranial Hypertension as First Manifestations of Multiple Endocrine Neoplasia Type 1

**DOI:** 10.3389/fendo.2019.00582

**Published:** 2019-08-28

**Authors:** Naiara C. B. Dantas, Carlos E. L. Soares, Manoel R. A. Martins, Delmar M. Lourenço, Ana R. P. Quidute

**Affiliations:** ^1^Walter Cantídio University Hospital, Federal University of Ceará, Fortaleza, Brazil; ^2^Faculty of Medicine, Drug Research and Development Center (NPDM), Federal University of Ceará (UFC), Fortaleza, Brazil; ^3^Endocrine Genetics Unit (LIM-25), Endocrinology Division, Hospital das Clínicas, School of Medicine, University of São Paulo, São Paulo, Brazil; ^4^Endocrine Oncology Division, Institute of Cancer of the State of São Paulo, São Paulo, Brazil

**Keywords:** giant prolactinoma, dopaminergic agonist, pituitary adenoma, obstructive hydrocephalus, intracranial hypertension, multiple endocrine neoplasia type 1

## Abstract

**Context:** Overall, giant prolactinomas are rare tumors (4%), especially those larger than 60 mm (1%). Despite the predominance of macroadenoma documented in multiple endocrine neoplasia type 1 (MEN1)-related prolactinoma, only three giant prolactinoma cases were described so far (size > 40 mm and prolactin > 1,000 ng/mL). None of them was larger than 60 mm or presented hydrocephalus or intracranial hypertension (ICH) as initial manifestation of MEN1.

**Case Description:** A 21-years-old man presented with ICH as the first clinical manifestation of MEN1. He harbored a *MEN1* germline mutation but refused periodic vigilance after normal hormonal screening at age 14 years. During investigation, magnetic resonance imaging (MRI) of the skull showed an expansive sellar/parasellar lesion (75 × 44 × 36 mm) with moderate to severe supratentorial obstructive hydrocephalus and an extremely high serum prolactin (PRL) of 10,800 ng/mL, without combined hypersecretion of other pituitary hormones. He was diagnosed with giant prolactinoma, and cabergoline was initiated. The patient evolved with early improvement of clinical complaints for hydrocephalus and ICH and PRL reached normal values (11 ng/mL) in association with significant tumoral shrinkage after 18 months on cabergoline. After 2 months of cabergoline, cerebrospinal fluid leakage was diagnosed and corrective surgery was provided. The mean dose of cabergoline was 3 mg/week throughout treatment.

**Conclusion:** We reported the first case with hydrocephalus and ICH as the initial clinical manifestation of a giant prolactinoma in MEN1. From our knowledge, this is the largest MEN1-related prolactinoma reported so far. Notably, all four MEN1-related giant prolactinomas cases reported were younger than 21 years strengthening the importance to routine *MEN1* genetic testing for prolactinoma in this age group. Also, they all had initial effective response with dopamine agonist ensuring this drug as first-line treatment for MEN1-related giant prolactinoma. However, the scarce number of treated patients and progression of cabergoline resistance in two of them suggest strict surveillance.

## Introduction

Multiple endocrine neoplasia type 1 (MEN1) is a rare autosomal dominant disease caused by inactivating germline mutations of the *MEN1* tumor suppressor gene that predisposes to the development of diverse endocrine and non-endocrine neoplasias. Tumors in parathyroid and pituitary glands and in duodenal/pancreatic endocrine cells are the most prevalent in MEN1 (1–3).

*MEN1* mutations carriers invariably present with primary hyperparathyroidism, resulting in a complete penetrance at age 50 years (2, 3). The non-functioning pancreatic neuroendocrine tumors are highly prevalent and are the main culprits for MEN1-related morbidity and mortality (4). A widely variable frequency of pituitary tumors has been reported (15–65%) in MEN1 patients (1, 5–7). Prolactinoma is the most prevalent tumoral subtype, accounting for ~60% of cases with MEN1-related pituitary adenomas (1, 5, 7). As in sporadic cases, MEN1-related prolactinoma is more prevalent in women (5–7), being frequently diagnosed during the fourth decade of life (1, 5–7).

Overall, prolactinomas are usually classified according to tumor size, as microprolactinomas (<10 mm in its largest diameter) or macroprolactinomas (≥10 mm). Most tumors (>70%) are slow growing microprolactinomas frequently found in women of childbearing age. In turn, macroprolactinomas are predominantly represented by tumors with dimensions of <40 mm, more frequently occurring in men and older women. In addition, macroprolactinomas larger than 40 mm, known as giant prolactinomas, are exceptionally rare, accounting for 0.5–4% of all prolactin-hypersecreting adenomas (8–10).

Giant prolactinomas are defined by combined association of the following features: largest diameter measuring 40 mm or more, significant extrasellar extension, hyperprolactinemia predominantly higher than 1,000 ng/ml, and absence of other pituitary hormone co-secretions, as GH and ACTH. Contrasting with microprolactinomas, giant prolactinomas are exceedingly more prevalent in men (9:1) (8–10). Despite the high prevalence of suprasellar extension and frequent visual impairment in giant prolactinomas, compressive symptoms associated with hydrocephalus and intracranial hypertension are very rare (8, 9).

Here, we described the profile of the complete tumoral and hormonal response to cabergoline, a dopaminergic agonist (DA), in a young man presenting with moderate hydrocephalus and intracranial hypertension caused by a giant prolactinoma as the first clinical manifestation of MEN1.

## Case Description

A 21-years-old man, as an at-risk member of a known MEN1 family, was initially invited to participate in a periodic clinical screening at age 14 years and 5 months. At that time, he had no complaints, and his pubertal development was normal. Routine biochemical and hormonal exams for MEN1 revealed no abnormalities, including pituitary hormones. At that time, sella turcica image was not initially performed. Since the patient did not adhere to the recommendations for annual assessment, he only sought medical care in the current situation, presenting with severe headache, nausea, vomiting and decreased visual acuity over 2 months. Additionally, he complained of sexual impotence and decreased libido. On physical examination, there was bilateral gynecomastia without galactorrhea and presence of hair rarefaction in axillary, pubic and facial regions.

**Figure 1:**
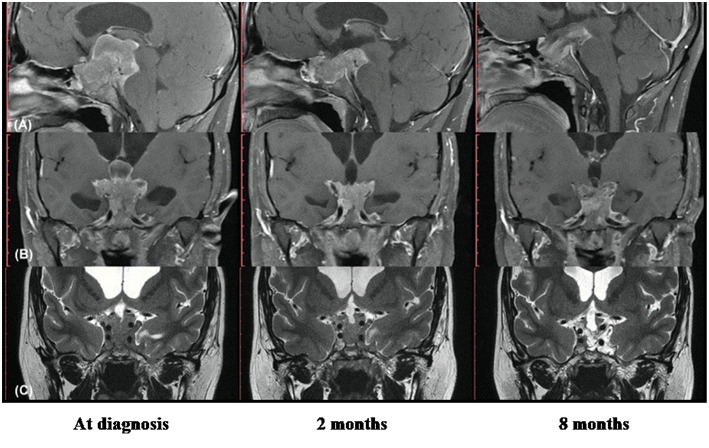
Radiological images showing remarkable therapeutic response with cabergoline in young man with hydrocephalus and intracranial hypertension for giant prolactinoma as first clinical manifestation of multiple endocrine neoplasia type 1. **(A)** Magnetic resonance imaging (MRI) in T1 with gadolinium, sagittal section; **(B)** MRI in T1 with gadolinium, coronal section; **(C)** MRI in T2 with gadolinium, coronal section. Left images show the solid-cystic sellar and parasellar tumoral mass identified at diagnosis (7.5 × 4.4 × 3.6 cm), with invasion of cavernous sinus, midbrain and third ventricle, occluded foramen of Monro and moderate to severe supratentorial obstructive hydrocephalus; Central images obtained 2 months after the beginning of cabergoline shows significant reduction of the tumor. Right images, with 8 months of cabergoline reveal a notable tumoral shrinkage and necrosis of the tumor.

He was admitted for diagnostic investigation. Magnetic resonance imaging of the skull revealed an expansive solid-cystic sellar and parasellar lesion measuring 75 × 44 × 36 mm, with no signs of calcification and no radiological evidence of suspected tumor hemorrhage. The tumoral mass invaded the cavernous sinus bilaterally, insinuating to the midbrain posteriorly, compressing the third ventricle and the foramen of Monro superiorly, and causing moderate to severe supratentorial obstructive hydrocephalus (Figure 1). The laboratory investigation revealed a very high serum level of diluted prolactin (PRL) of 10,800 ng/mL (reference value: 2.5–17 ng/mL) with no co-secretion of other pituitary hormones, thus compatible with the biochemical diagnosis of giant prolactinoma. Investigation for deficiencies of pituitary axes was normal, except for a hypogonadotropic hypogonadism (Table 1). Campimetry did not show any visual stimuli (black field) in the right eye and revealed diffuse loss of sensitivity and scotomas in the left eye.

**Table 1:** Outcome of pituitary hormones during first-line treatment with dopaminergic agonist of MEN1 case with giant prolactinoma.

**Hormones**	**At diagnosis**	**20 days**	**1 month**	**2 months**	**3 months**	**6 months**	**9 months**	**12 months**	**18 months**
Diluted prolactin (2.5–17 ng/mL)	10,800	1,315	688	150	150	56.4	27	22	11
Total testosterone (262–1,593 ng/dL)	186	–	–	186	–	327	220	228	277
FSH (0.7–11.1 mUI/mL)	2.3	–	–	2.3	–	–	–	–	–
LH (1.1–11.06 mUI/mL)	3.1	–	–	3.1	–	–	–	–	–
TSH (0.4–4.0 μIU/mL)	0.92	–	–	0.77	–	0.6	–	–	–
Free T4 (0.89–1.76 ng/dL)	1.13	–	–	1.54	0.86	1.04	–	–	1.08
IGF-1 (116–358 ng/mL)	211	–	–	–	186	–	–	–	110^*^
Basal cortisol (5–25 μg/dL)	9.27	–	–	11	10	12	–	–	17
CAB (mg/week)	–	1	1.5	1.5	1.5	3	3	3	3

*FSH, follicle-stimulating hormone; LH, luteinizing hormone; TSH, thyroid-stimulating hormone; CAB, cabergoline*.

* *Normal range of IGF1 values between 22 and 24 years (99.7–289 ng/mL)*.

Cabergoline was initiated at a dose of 0.5 mg twice a week after a multidisciplinary decision including the neurosurgery team. Initially, it was decided to close surveillance attempting to avoid emergency invasive surgical procedures, such as external ventricle drainage. In the subsequent days of hospitalization, therapy with cabergoline appeared fully effective, as progressive and sustained improvement of headache, nausea, and vomiting was reported. After 12 days on cabergoline treatment, the patient was discharged still with visual alteration but with complete improvement of the intracranial hypertension symptoms.

In clinical follow-up, after 1 month of treatment, the serum PRL was 1,315.8 ng/mL, and after 2 months, it dropped to 150 ng/mL. Clinically, there was a marked visual improvement in the left eye. However, the patient complained of abundant fluid in the nasal cavity; rhinorrhea was promptly confirmed and a surgical procedure for correction of cerebrospinal fluid leakage was provided. Prior to surgery, MRI revealed a tumoral lesion reduction (45 × 35 × 26 mm) with no radiological signs of local bleeding. The tumor extended posteriorly to the pre-mesencephalic/pontine cistern in close contact with the third ventricle floor and there was moderate dilation of the supratentorial ventricular system, with resolution of hydrocephalus (Figure 1).

After 9 months on cabergoline therapy, serum PRL values (27 ng/mL) were mildly elevated and became normal with 18 months (Table 1). The pituitary MRI showed a marked tumor shrinkage. It became predominantly cystic with areas of necrosis beyond complete resolution of the supratentorial ventricular system dilation and significant reduction of the intraventricular tumoral component (Figure 1).

Despite the effective hormonal control (PRL, 22.1 ng/mL) and remarkable tumoral reduction after 12 months of treatment with cabergoline, total testosterone was low (228 ng/mL, normal values: 262–1,593 ng/mL) and complaints of hypogonadism remained. Thus, hormone replacement therapy with testosterone was initiated. After 18 months, at a mean dose of cabergoline of 3 mg/week, the patient was asymptomatic and PRL levels remained within the normal range (11.1 ng/mL). In addition, the periodic radiological and hormonal screening for MEN1-related tumors was performed during follow-up, allowing the diagnosis of asymptomatic primary hyperparathyroidism (PHPT) and absence of adrenocortical or pancreatic neuroendocrine tumors.

**Figure 2:**
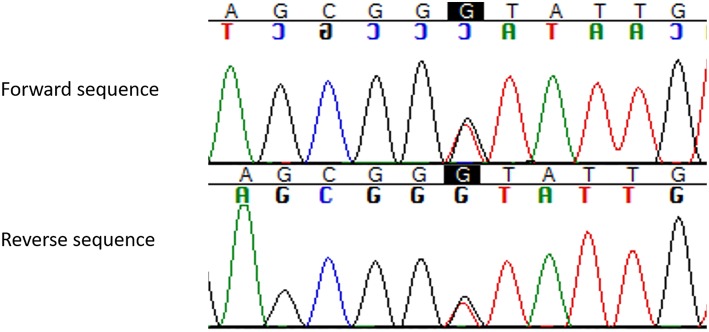
Pathogenic germline *MEN1* variant (c.654 + 1G > T, IVS3, g.3405G > T) identified in the present MEN1 case reported with hydrocephalus and intracranial hypertension for giant prolactinoma. The change in heterozygous of the nucleotide guanine for timine at the canonic region +1 of the intron 3 of the *MEN1* gene (c.654 + 1G > T; HGMD: CS982266; dbSNP: rs794728622) results in a splicing donor variant (ref. seq: ENST00000312049; NM_130799).

As expected, the genetic testing documented the same splice site mutation (IVS3, c.654 + 1G > T) found in the index case and in other affected family members (Figure 2).

## Discussion

We reported the peculiar case of a young man with MEN1 syndrome presenting with a combination of rare or exceptionally rare events, such as presence of giant prolactinoma larger than 6 cm; pituitary tumor as the first clinical manifestation of MEN1 and occurrence of hydrocephalus and intra cranial hypertension, as initial symptoms of giant prolactinoma.

Sporadic (non-familial) giant prolactinomas are rare tumors representing, respectively, 0.5% of all pituitary tumors and 4% of all prolactinomas, as reviewed by Maiter and Delgrange (8). Most of them are characterized by tumors smaller than 6 cm. In fact, only few cases whose dimensions exceed this diameter have been described either as part of several small series of giant prolactinomas or as case reports (8, 11, 12). So far, only one study focused on this specific tumor subset and compiled 18 cases with giant prolactinomas larger than 60 mm (9). The latter cases accounted for no more than 1% of all prolactinoma patients from five tertiary reference centers reported in a period of 20 years, highlighting the marked rareness of this condition (9). To our knowledge, we reported the first patient with MEN1 syndrome presenting with a giant prolactinoma larger than 6 cm (Table 2).

**Table 2:** Main genetical and clinical findings and outcome of four young patients with MEN1-related giant prolactinoma.

**Reference**	Age^*^ (years)/Sex	**G-PRLoma as first clinical manifestation/hydrocephalus/other symptoms**	**Prolactin (ng/ml)**	**Tumor maximal diameter (mm)**	**Altered pituitary axes**	**Index case/number of *MEN1*-positive family members with index-case included)**	*MEN1* genetic analysis^***^	**Medication (doses)**	**Cabergoline response**	**Surgery for tumor**	**Other MEN1 associated tumors**
Subasinghe et al. (13)	8/M	Y/N/ Headache Visual disturbance	91,800	59	–	Y/3	c.781C **>** T; p.Gln261^*^ (NS); exon 4	Cabergoline (7 mg/week)	Resistant, but with initial significant response	Transcranial excision, radiotherapy	Insulinoma PHPT
Gan et al. (14)	11/M	Y/N/ Headache Visual disturbance	1,114	55	GH, TSH	Y/7	c.784 – 9G > A (SS); intron 4	Cabergoline (3.5 mg/week)	Resistant, but with initial significant response	Transcranial excision, radiotherapy	PHPT
Subasinghe et al. (13)	20/F	Y/N/ Galactorrhoea Secondary amenorrhoea	8,930	45	–	Y/3	c.1736T > C; p.Leu579Pro (MS); exon 10	Cabergoline (3.5 mg/week)	Tumor shrinkage	No	PNET PHPT
Present case	21/M	Y/Y/ Headache Visual disturbance Decreased libido	10,800	75	FSH, LH	N^**^/4	c.654 + 1G > T (SS); intron 3	Cabergoline (3 mg/week)	Tumor shrinkage	No	PHPT

*A, adenine; C, cytosine; F, female; FSH, follicle-stimulating hormone; G, guanine; GH, growth hormone; IVS, intervening sequence; LH, luteinizing hormone; M, male; MEN1, multiple endocrine neoplasia type 1; MEN1, MEN1 gene; mm, millimeter; MS, missense; N, no; NS, nonsense; PHPT, primary hyperparathyroidism; PNET, non-functional pancreatic neuroendocrine tumor; PRL prolactin; SS, splice site mutation; T, thymine; TSH, thyroid-stimulating hormone; Y, yes*.

* *Age at the MEN1 diagnosis*.

** *Screened patient since the age of 14, but he lost the follow-up*.

*** *MEN1 reference sequence chosen was NM 130799.2*.

Despite the small number of MEN1-related macroprolactinoma cases in patients younger than 21 years (14, including the present case) (13–20), the frequency of giant prolactinomas at this age group is much higher (4/11; 36%) than the overall estimated prevalence (2–4%) of age-independent giant prolactinomas (8–10). These data may suggest that in younger MEN1 patients the frequency of giant prolactinomas is higher than in age-matched sporadic cases. Overall, giant prolactinomas are frequently diagnosed at the fourth to fifth decades being very rare in children and adolescents (8, 13). Thus, most of the 140 giant prolactinoma cases compiled from a meta-analysis were diagnosed around 40 years of age. Furthermore, only 16 cases with giant prolactinoma were identified during infancy and adolescence (8), highlighting the rarity of the present case report.

Three large series including apparently sporadic pituitary tumors diagnosed at younger ages have recommended routine genetic screening for *MEN1* and *AIP* genes (19–21). Despite the occurrence of MEN1-related macroprolactinoma in 5% of patients younger than 20 y (3/59) (20) and 10% (3/30) in those younger than 18 years (19), there was no case with MEN1-related giant prolactinoma. Several other prolactinoma series at young ages have been reported either with no *MEN1* genetic analysis (22–26) or MEN1 cases eventually diagnosed due to familial history and/or molecular diagnosis (27), suggesting potential underdiagnosis of this condition in this age group.

So far, four MEN1-related giant prolactinomas were diagnosed at young ages varying from 8 up to 21 years (present case included) (Table 2). Importantly, giant prolactinoma was the initial clinical manifestation of MEN1 in all four cases (Table 2). Pituitary adenoma may be the first clinical manifestation in up to 11–21% of the cases diagnosed with MEN1 syndrome (4, 5, 15, 16). Our patient had prolactinoma as the first MEN1-related tumor, developed during the end of the second decade of life, as puberty, growth acceleration and pituitary hormones were normal at age 15 years. The three cases previously reported (tumors <60 mm) were apparently sporadic. However, a *MEN1* germline mutation was underscored and genetic screening allowed detection of newly affected family cases in all of them (Table 2). These data strengthen the importance to routine *MEN1* genetic testing for prolactinoma in this age group, as suggested in more recent series (19, 20). Conversely, our case was a family member of a known MEN1 genealogy, although he lost the opportunity of early diagnosis, not adhering to periodic screening. The adoption of inappropriate coping mechanism especially of denial and cognitive avoidance of the disease is common in inherited cancer syndromes, as seen in our case (28).

The main clinical manifestations associated with giant prolactinomas are accounted for hypogonadism (61% of cases), vision alteration (71%) and recurrent headache (59%) (8), as documented in our case (Table 1). Hydrocephalus is very rarely associated with giant prolactinomas and there are debates on its therapeutic management (8, 9, 29–38).

Four cases with giant prolactinomas underwent transsphenoidal surgery for intracranial hypertension and giant macroadenomas in a series of 18 patients with prolactinomas larger than 60 mm (9). In turn, Chentli et al. described three cases of obstructive hydrocephalus in a series of 44 patients (6.8%) with giant prolactinomas (30). The very low prevalence of hydrocephalus and intracranial hypertension in giant prolactinomas could be resultant of very slow tumor growth (30). Again, *MEN1* genetic analysis was not available and no MEN1 case was diagnosed in both series (9, 30). Most cases of giant prolactinoma with hydrocephalus were diagnosed in young males, as seen in our case (29, 31, 32, 34–38). However, in a recent series of 23 men with giant prolactinomas, no one had hydrocephalus (10). Labauge et al. described a MEN1 case with intracranial hypertension after hemorrhagic necrosis of the tumoral lesion, contrasting with our case (39).

In a recent review, 140 giant prolactinoma cases from 15 small series ranging from 4 to 20 patients were selected. Surprisingly, no MEN1 case was reported in this large series (8). Reviewing a large French series of 77 young cases (<20 years) with macroprolactinoma that were investigated to detection of AIP and MEN1 germline mutations, we actively identified 10 cases (14%; 10/71) meeting the diagnostic criteria for giant prolactinoma aging 10–19 years (20), but none of them was MEN1-related giant prolactinoma (20). Again, these data emphasize the rarity of case presently reported.

Overall, there were three cases with MEN1-related macroprolactinoma (5%; 3/77) measuring, respectively, 30, 32, and 50 mm in this French series (20). Based on criteria adopted for most series and authors (8–10, 12–14, 40–45), this latter case was not defined as giant prolactinoma as prolactin was lower than 1,000 ng/ml. However, this is controversial as other authors has considered lower prolactin cut-offs as 200 or 250 ng/ml to define giant prolactinoma (30, 46–48).

Moraes et al. (48) and Maiter and Delgrange (8) emphasized the importance of exclude hook effect when there is dissociation between prolactin values and tumor volume in giant prolactinoma. Also, they reinforced that tumoral hemorrhagic or cystic component may lead to lower values of prolactin than expected. Unfortunately, these concerns are not informed in most papers (8, 34, 48) and it is possible that cup of cases with true giant prolactinoma but with prolactin values lower are underdiagnosed or erroneously diagnosed as non-giant macroprolactinoma or even as non-functioning pituitary macroadenomas (8, 48). Thus, to that case with MEN1-related macroprolactinoma (size, 5.0 cm; prolactin, 512 ng/ml) (20), it is not possible to exclude definitely a giant prolactinoma as there were data if the tumor had cystic component or if dilutions of prolactin were supported (20).

In a large MEN1 series, macroadenomas were two times more frequent in MEN1-related tumors than in sporadic non-MEN1 prolactinomas (85 vs. 42%) (5). In addition, clinical signs related to tumor size and poor response to DA therapy were more frequently observed in this MEN1 cohort than in non-MEN1 subjects (58 vs. 10%) (5). However, in a recent study including *MEN1* mutation-positive carriers from known MEN1 families, a high response rate (90%) to DA therapy was documented in a subset of MEN1-related prolactinoma, independent of the tumor's size (6).

The fast tumoral growth observed in our case, reaching 75 mm suggested a potentially higher tumor aggressiveness. This possibility was reinforced by the early occurrence of intracranial hypertension at age 21 years, which might reduce responsiveness to DA. However, an excellent therapeutic response was documented in our case, as seen by the fast improvement of symptoms, complete resolution of obstructive hydrocephalus and normalization of serum prolactin (Table 1) followed by remarkable tumoral shrinkage and necrosis (Figure 1).

Worthwhile, highly positive responses to DA were also reported in most giant prolactinoma cases, as evidenced by normalization of prolactin (60%) and tumor response (74%) (8). Similar results were obtained in prolactinomas larger than 60 mm (9). Also, remarkable therapeutic responses to DA were recently reported in 12 giant prolactinoma male cases (10). Based on these data from sporadic giant prolactinomas, DA was primary therapeutic choice in all the four reported cases with MEN1-related giant prolactinoma. Initially, all four cases had an effective tumoral and hormonal response after DA administration, including resolution of intracranial hypertension in our case. In the follow up, two of them remained highly DA responsive. In the two other cases, resistance occurred after an initial effective response. Despite of that, selection of DA as first-line treatment in prolactinoma/MEN1 cases seems highly plausible (Table 2).

Accordingly, DA is the treatment of choice also in cases with giant prolactinoma and obstructive hydrocephalus (8), as reinforced by our case. Thus, invasive surgical procedures should be initially avoided. Such interventions as external ventricular drainage and transsphenoidal or transcranial surgeries have been reserved for specific patients who evolve with cerebrospinal fluid leakage, apoplexy, intolerance, and insufficient tumor response or tumor progression during treatment with DA (8–10). Nevertheless, the small number of cases with MEN1-related giant prolactinomas and the development of drug resistance secure strict vigilance.

In most cases, DA administration need to be continued even after surgery. Giant prolactinomas >60 mm have been treated with initial doses of cabergoline of 1–1.5 mg/week, staggered every 2–4 months, with a mean therapeutic dose of 3.5 mg/week that effectively reduced the levels of prolactin (9). There is no recommendation to start with high doses of DA or to increase them quickly as some patients may have an early, prompt and highly efficient therapeutic response with normalization of prolactin and tumoral shrinkage, which favors the cerebrospinal fluid leakage onset or apoplexy (8, 49). Accordingly, our patient started at a dose of 1 mg/week, with a slow increase up to 3 mg/week. Despite the gradual dose increase, cerebrospinal fluid leakage was noticed 2 months after starting treatment, requiring surgical correction.

The splice site mutation c.654 + 1G > T harbored by our case was first reported by Teh et al. (50). Of note, we have previously reported a sporadic MEN1 case (51, 52) and more recently other nine apparently unrelated familial MEN1 cases harboring this mutation (53). Due to its very low frequency, the *MEN1* c.654 + 1G > T mutation is not included within the nine “warm spot” *MEN1* variants (54). Thus, a founder mutation could be hypothesized. In this line, our case and his family could be unrecognized members of one of these 10 MEN1 Brazilian families early reported (53). Genealogy expansion and haplotype studies may potentially elucidate this question.

## Conclusion

We reported, to our knowledge, the first MEN1-related prolactinoma larger than 60 mm and in which the first clinical manifestations were hydrocephalus and intracranial hypertension. So far, only three previous MEN1-related prolactinoma cases were reported as giant prolactinoma (tumors < 60 mm). Notably, in all four cases, the giant prolactinoma was the first clinical manifestation, and the diagnosis/first symptoms occurred during the first two decades of life. These data reinforce previous studies indicating *MEN1* screening for young patients with macroprolactinoma. Despite the paucity of cases, first-line treatment with DA should be recommended for MEN1-related giant prolactinoma, as all reported cases had at least an initially effective and positive response of tumoral and/or hormonal control, including rapid resolution of emergency compressive neuro-ophthalmological symptoms due to hydrocephalus and intracranial hypertension, as seen in our case. In addition, close surveillance should be provided for the risk of cerebrospinal fluid leakage or apoplexy or resistance during DA therapy, as recommended for sporadic tumor counterparts.

## Ethics Statement

This study was carried out in accordance with the recommendations of the Ethical Committees from both Institutions involved with written informed consent from patient reported. The patient gave written informed consent in accordance with the Declaration of Helsinki. The protocol was approved by the Ethical Committee (CAPPesq) of the University of São Paulo and by Ethical Committee of the Federal University of Ceará.

## Author Contributions

AQ designed the study. ND and CS collected the data and created the database. AQ, MM, and DL analyzed the data. ND, AQ, and DL wrote the manuscript. MM and AQ followed the patient. All authors contributed to manuscript revision, read, and approved the submitted version.

### Conflict of Interest Statement

The authors declare that the research was conducted in the absence of any commercial or financial relationships that could be construed as a potential conflict of interest.
